# Intensive DNA Replication and Metabolism during the Lag Phase in Cyanobacteria

**DOI:** 10.1371/journal.pone.0136800

**Published:** 2015-09-02

**Authors:** Satoru Watanabe, Ryudo Ohbayashi, Yu Kanesaki, Natsumi Saito, Taku Chibazakura, Tomoyoshi Soga, Hirofumi Yoshikawa

**Affiliations:** 1 Department of Bioscience, Tokyo University of Agriculture, Tokyo, Japan; 2 Core Research for Evolutional Science and Technology (CREST), Japan Science and Technology Agency (JST), Saitama, 332-0012, Japan; 3 Genome Research Center, Tokyo University of Agriculture, Tokyo, Japan; 4 Institute for Advanced Biosciences, Keio University, Tsuruoka, Yamagata, Japan; Mount Allison University, CANADA

## Abstract

Unlike bacteria such as *Escherichia coli* and *Bacillus subtilis*, several species of freshwater cyanobacteria are known to contain multiple chromosomal copies per cell, at all stages of their cell cycle. We have characterized the replication of multi-copy chromosomes in the cyanobacterium *Synechococcus elongatus* PCC 7942 (hereafter *Synechococcus* 7942). In *Synechococcus* 7942, the replication of multi-copy chromosome is asynchronous, not only among cells but also among multi-copy chromosomes. This suggests that DNA replication is not tightly coupled to cell division in *Synechococcus* 7942. To address this hypothesis, we analysed the relationship between DNA replication and cell doubling at various growth phases of *Synechococcus* 7942 cell culture. Three distinct growth phases were characterised in *Synechococcus* 7942 batch culture: lag phase, exponential phase, and arithmetic (linear) phase. The chromosomal copy number was significantly higher during the lag phase than during the exponential and linear phases. Likewise, DNA replication activity was higher in the lag phase cells than in the exponential and linear phase cells, and the lag phase cells were more sensitive to nalidixic acid, a DNA gyrase inhibitor, than cells in other growth phases. To elucidate physiological differences in *Synechococcus* 7942 during the lag phase, we analysed the metabolome at each growth phase. In addition, we assessed the accumulation of central carbon metabolites, amino acids, and DNA precursors at each phase. The results of these analyses suggest that *Synechococcus* 7942 cells prepare for cell division during the lag phase by initiating intensive chromosomal DNA replication and accumulating metabolites necessary for the subsequent cell division and elongation steps that occur during the exponential growth and linear phases.

## Introduction

Cell physiology during the exponential and stationary phases of bacterial growth has been studied extensively [1]. During exponential growth, cells undergo multiple rounds of DNA replication, accompanied by cell division, resulting in rapid cell proliferation. As a consequence of prolonged starvation, the cells enter stationary phase, during which proliferation and metabolic activity is markedly reduced to maintain viability under nutrient-limiting conditions. Lag phase begins when stationary-phase cells encounter new sources of nutrients. During this phase, cells recover from the nutrient-limiting conditions and begin to synthesize the cellular components necessary for outgrowth [2]. Former studies of monoploid bacteria, such as *Escherichia coli*, have shown that dilution of a stationary-phase culture into fresh minimal medium transiently synchronizes cell cycle events, including DNA replication, cell division, and cellular metabolism [3–5]. The synchronicity indicates that DNA replication is strictly coupled to chromosome segregation and cell division in monoploid bacteria [6, 7].

Cyanobacteria are the predominant phototrophs in ocean and freshwater ecosystems, and they represent one of the oldest and most widespread phylogenetic clades. Because these species have oxygen-producing photosynthetic capabilities, there is increasing interest in using cyanobacteria to convert solar energy into biomass. While marine cyanobacteria, such as *Prochlorococcus* sp. and *Synechococcus* sp., harbour a single circular chromosome [8, 9], several species of freshwater cyanobacteria, including *Synechococcus elongatus* PCC 7942 and *Synechocystis* sp. PCC 6803 (hereafter *Synechococcus* 7942 and *Synechocystis*, respectively), harbour multiple chromosomes per cell; they are therefore considered polyploid organisms [8, 10–12]. Similar polyploidy in the genomic copy number is observed in the plastids of plants [13, 14], which are thought to derive from cyanobacterium.

In cyanobacteria, growth is dependent on both the nutrient composition of the medium and light intensity. In a batch culture, cyanobacterial growth progresses from a lag phase into an exponential growth phase. This is typically followed by a period of arithmetic (linear) growth phase that continues until the culture reaches a non-growing stationary phase [15]. In cyanobacteria, linear growth is most often associated with light limitation caused by self-shading of cells as cultures reach a certain cell density [16–18]. Although there are several studies on growth phase-dependent responses in cyanobacteria [16, 17, 19–21], the relationship between growth phase and chromosome copy number is still poorly understood.

In previous studies, we examined the replication mechanism of *Synechococcus* 7942. *Synechococcus* 7942 exhibits light-dependent growth and DNA replication [8, 22]. Using next-generation sequencing and quantitative real-time PCR techniques, we confirmed the predicted origin of replication (*ori*) region and the progression of chromosomal replication [23]. DNA replication proceeds bi-directionally from the *ori* region in *Synechococcus* 7942, indicating that the fundamental mechanisms of DNA replication, including initiation and progression, in *Synechococcus* 7942 are similar to those in single copy bacteria, such as *E*. *coli* and *Bacillus subtilis*. However, we also found that replication in *Synechococcus* 7942 initiates asynchronously, not only among cell populations, but also among multi-copy chromosomes [23–25]. These findings suggest that DNA replication is not tightly coupled to cell division in *Synechococcus* 7942, unlike in single copy organisms.

In the present study, we address this issue by characterizing physiological characteristics at distinct growth phases. We assessed the chromosomal copy number during each growth phase. Additionally, we assayed the DNA replication activity in the lag phase relative to activity in subsequent phases and performed metabolomic analyses of *Synechococcus* 7942 cultures. Lastly, we investigated the mechanism by which the chromosome copy number is regulated at different growth phases.

## Materials and Methods

### Strains and culture conditions

*Synechococcus* 7942 wild-type and derivative strains were grown photoautotrophically at 30°C in the presence of bubbling 2% CO_2_ and continuous illumination (40 μmole photons·m^-2^·s^-1^) in modified BG11 medium [26] containing 35 mM NaNO_3_ (double the amount in normal BG11). To synchronize cell growth, cells were cultured in BG11 medium for 10 days until they reached to OD_750_ = 10 (stationary culture). They were then diluted to OD_750_ = 0.2 in fresh BG11 medium and incubated for 18 hr in the dark. To reinitiate cell growth, cultures were re-exposed to light conditions (time 0) and incubated in the presence of light for the remainder of the experiment. For the preparation of stationary culture of *Synechococcus* 7942^TK^, the medium was supplemented with spectinomycin at a final concentration of 40 μg/ml.

### Flow cytometry

Flow cytometry analyses were performed as previously described [23].

### BrdU incorporation assay

BrdU incorporation was evaluated by counting the number of BrdU-positive cells, according to the method of Watanabe et al. [23].

### Analysis of the composition of the BG11 growth medium

To analyse the composition of the BG11 growth medium, supernatant from *Synechococcus* 7942 culture grown in BG11 medium for 1 week was obtained by centrifugation and filtration. The composition (anions: nitrate, sulphate, chlorine, and phosphate; cations: sodium, potassium, calcium, magnesium, ferric and manganese) of fresh and spent BG11 media was analysed with the help of a commercial service (Yakult Central Institute, Tokyo, Japan).

### Measurement of inorganic phosphate content of the supernatant from *Synechococcus* 7942 batch culture

The inorganic phosphate content of the batch culture was assayed as follows: after centrifugation, the supernatant obtained from *Synechococcus* 7942 culture at each time point was subjected to a malachite green assay (BioAssay Systems Co., CA, USA) and the amount of inorganic phosphate was measured according to the manufacturer’s instructions.

### RNA blotting analysis

*Synechococcus* 7942 cultures were harvested at each time point by rapid filtration through hydrophilic polyethersulphonate filters (Pall Supor 800 Filter, 0.8 mm). The filter covered with cells was immediately immersed in 1 ml of PGTX solution [27] and frozen in liquid nitrogen. Total RNA was extracted and analysed by gel electrophoresis and northern blotting as described [28]. To generate RNA probes of *sphX* (ORF ID: Synpcc7942_2445) and *pstS* (Synpcc7942_2444), template DNAs were PCR amplified using the following primer sets: 2445_f: 5'- GGCTGTGATGCCTTCTGTCC-3', 2445_rT7: 5'- TAATACGACTCACTATAGGGAGATCAGTTGATGCCTTCAGC-3', 2444_f: 5'- GGAATCAGATCGATCCGAGCTTCC-3' and 2444_rT7: 5'- TAATACGACTCACTATAGGGAGAGACAAAGAGCGGCCGCG-3'

### Metabolomic analyses

Synchronized *Synechococcus* 7942 wild-type cells were harvested with fast filtration and immediately suspended in the extraction solution, as described previously [29]. Metabolite concentrations were analysed with capillary electrophoresis time-of-flight mass spectrometry (CE-TOFMS), using an Agilent CE capillary electrophoresis system equipped with an Agilent 6210 time-of-flight mass spectrometer, an Agilent 1100 isocratic HPLC pump, an Agilent G1603A CE-MS adapter kit, and an Agilent G1607A CE-ESI-MS sprayer kit with an attached platinum needle (Agilent Technologies, Waldbronn, Germany). Cationic metabolites were separated with a fused silica capillary, using 1 M formic acid as the electrolyte. Anionic metabolites and nucleotides were separated with a COSMO (+) capillary (Nacalai Tesque, Kyoto, Japan), using 50 mM ammonium acetate (pH 8.5) as the electrolyte. Details of the CE-TOFMS conditions and the methods used for data analysis were described previously [30]. Absolute quantifications were performed using metabolite standards and calculated as the amount of metabolite per 10^7^ cells (i.e. pmol per 1 × 10^7^ cells) in each sample (S2 Table). We were unable to separate the TOF-MS peaks of 3-phosphoglycerate (3-PG) and 2-phosphoglycerate (2-PG). Consequently, they are depicted as the combined total amounts of 3-PG and 2-PG.

## Results

### Characterization of growth phases in *Synechococcus* 7942 batch cultures

**Fig 1:**
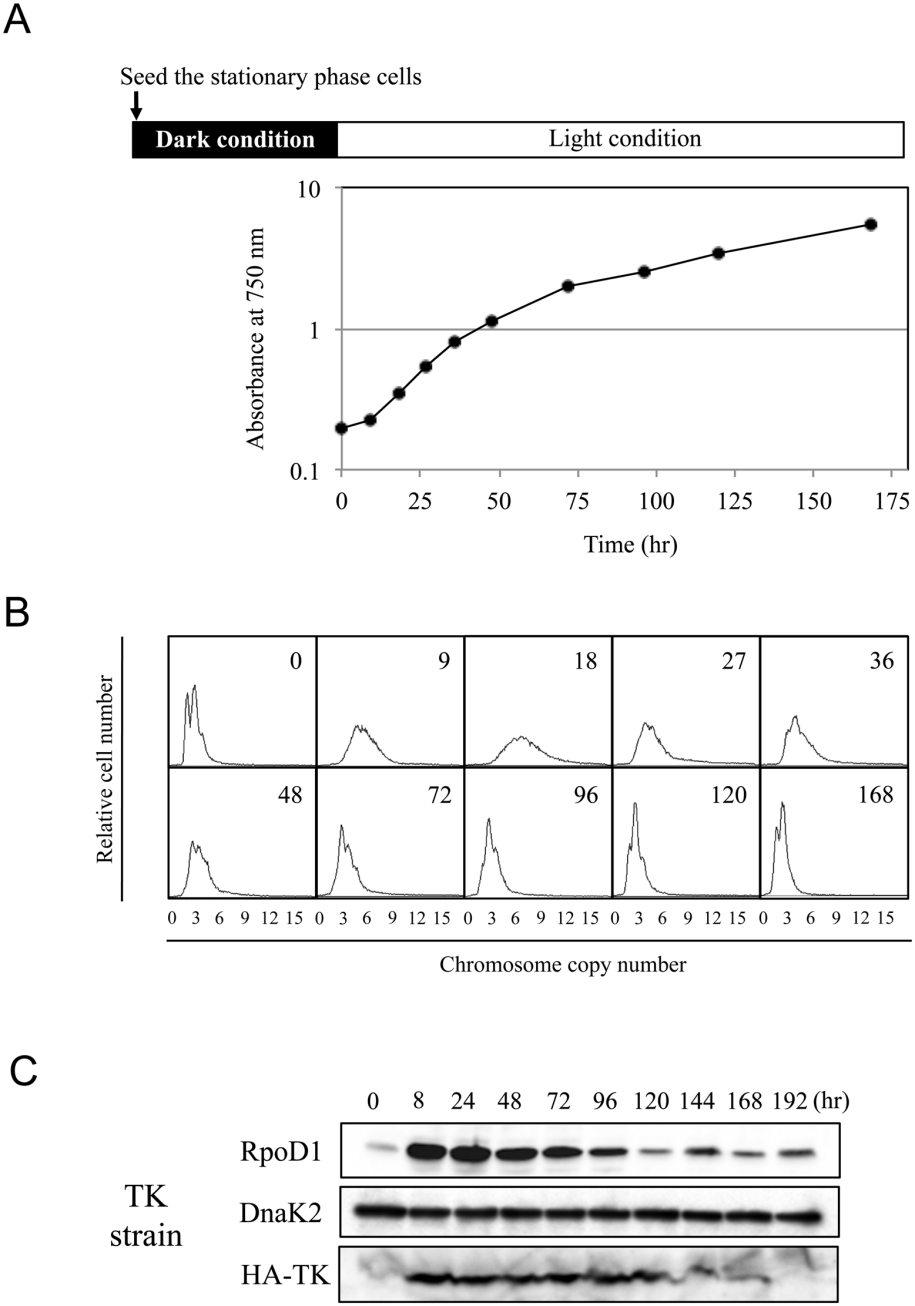
Growth and chromosome copy number during long-term culture. (A) Growth curve of *Synechococcus* 7942^TK^ after transfer to illuminated conditions, with a diagram showing the culture strategy. The stationary cultures were diluted to OD_750_ = 0.2 in fresh BG11 liquid medium. After 18 hr in the dark, cultures were transferred to light conditions (time 0). (B) DNA content profiles of *Synechococcus* 7942^TK^ cells. Cells were harvested at each time point and analysed with flow cytometry. (C) Expression of RpoD1, DnaK2, and HA-tagged thymidine kinase (HA-TK). *Synechococcus* 7942^TK^ cultures were harvested at the indicated times after transfer to liquid medium, and crude extracts were prepared. Samples containing 5 μg (RpoD1 and DnaK2) or 20 μg (HA-TK) of total protein were analysed with western blot using the appropriate antibodies.

In previous studies, we observed significant changes in the chromosomal copy number of *Synechococcus* 7942 in growth-synchronized cultures [23]. While the copy number decreased significantly during stationary phase, it increased when the cells were transferred to fresh BG11 medium and illuminated. To elucidate this phenomenon, we first studied the process of copy number change during long-term culture. A stationary culture of *Synechococcus* 7942^TK^, which contains an HA-tagged thymidine kinase (HA-TK) gene with which to assess the frequency of DNA replication, was diluted, incubated in the dark for 18 h, and then transferred to the light condition to restart cell growth. As depicted in Fig 1A, *Synechococcus* 7942^TK^ cells entered exponential growth approximately 18 hr after exposure to light. Growth gradually slowed after 48 hr and nearly arrested after 120 hr. We therefore defined the exponential phase as hours 18–48, and beginning of the linear phase as 48 hours post-light exposure.

The DNA content of the cells at each growth phase was analysed with flow cytometry. The starting culture contained 2–4 chromosomal copies per cell and exhibited a spike-shaped pattern (Fig 1B, Panel 0), consistent with cells that have completed a full round of DNA replication. Conversely, the DNA profile at 18 hr after transferring to the light condition (Fig 1B, Panel 18) manifested a broader shape, and the chromosomal copy number was significantly higher (4–10 copies per cell) than that in the starting culture. The profile gradually returned to a spiky shape at 48 hr post-transfer, and the copy number remained at approximately 2–4 copies per cell throughout the linear phase (Fig 1B, Panels 72–168). The reduction in chromosomal copy number continued during the late stage of linear phase, eventually returning to 2–4 copies per cell by 96 hr (Fig 1B, Panels 96–168).

We next analysed the expression patterns of HA-TK and two essential proteins, RpoD1, the principal sigma factor [31, 32], and DnaK2, an Hsp70 family chaperone protein involved in the stress response [33, 34]. The expression of RpoD1 and HA-TK was markedly increased in the exponential phase (Fig 1C, 8–24 hr after inoculation), compared to expression in the starting culture. Levels then decreased during the linear phase (48–192 hr), indicating that RpoD1 is primarily involved in the regulation of genes necessary during the exponential growth of *Synechococcus* 7942. Conversely, DnaK2 was expressed at similar levels at each time point (Fig 1C). The Hsp70/DnaK chaperone family is required to prevent aggregation and maintain proper folding of many proteins. Therefore, it is likely that consistent expression of DnaK2 proteins is essential during all growth phases.

We quantified the major components of the liquid BG11 medium before and after 1 week of culturing *Synechococcus* 7942. Compared to the fresh BG11 medium, phosphate and ferric ions were almost lost in the supernatant of 1 week-old culture (S1 Table). In addition, potassium ion content of the 1-week-old culture was found to be less than half of that of fresh BG11. Since phosphate is a primary substrate for DNA synthesis, we additionally assayed the amount of inorganic phosphate under batch culture conditions. The phosphate content gradually decreased with the culturing time and reached an almost undetectable level 96 hr post-transfer (S1 Fig). To assess the cellular response of *Synechococcus* 7942 to phosphate-limitation in batch culture conditions, we investigated the expression of the phosphate binding proteins (*sphX*: Synpcc7942_2445 and *pstS*: Synpcc7942_2444), which are subunits of the ABC-type phosphate transporter and induced in *Synechocystis* sp. PCC 6803 under phosphate-limiting conditions [35, 36]. The levels of both *sphX* and *pstS* transcripts transiently increased during the lag phase (9 hr post-transfer), and were dramatically upregulated in the late linear phase (72 hr and 120 hr post-transfer) (S1 Fig). This indicates that the expression of the phosphate transporter was strongly induced when the amount of phosphate in the BG11 growth medium was decreased to less than 15% of that in the fresh medium (S1 Fig, 72 hr post-transfer). At the beginning of the linear phase (48 hr post-transfer), the induction of phosphate-binding subunits was not observed, suggesting that the phosphate level at the time (more than 70% of that of fresh BG11 medium) was sufficient for cell survival and growth (S1 Fig, 48 hr post-transfer). These results suggest that phosphate limitation is not a major factor in triggering the switching from exponential phase to linear phase.

### Comparison of DNA replication frequency in the lag and exponential phases

**Fig 2:**
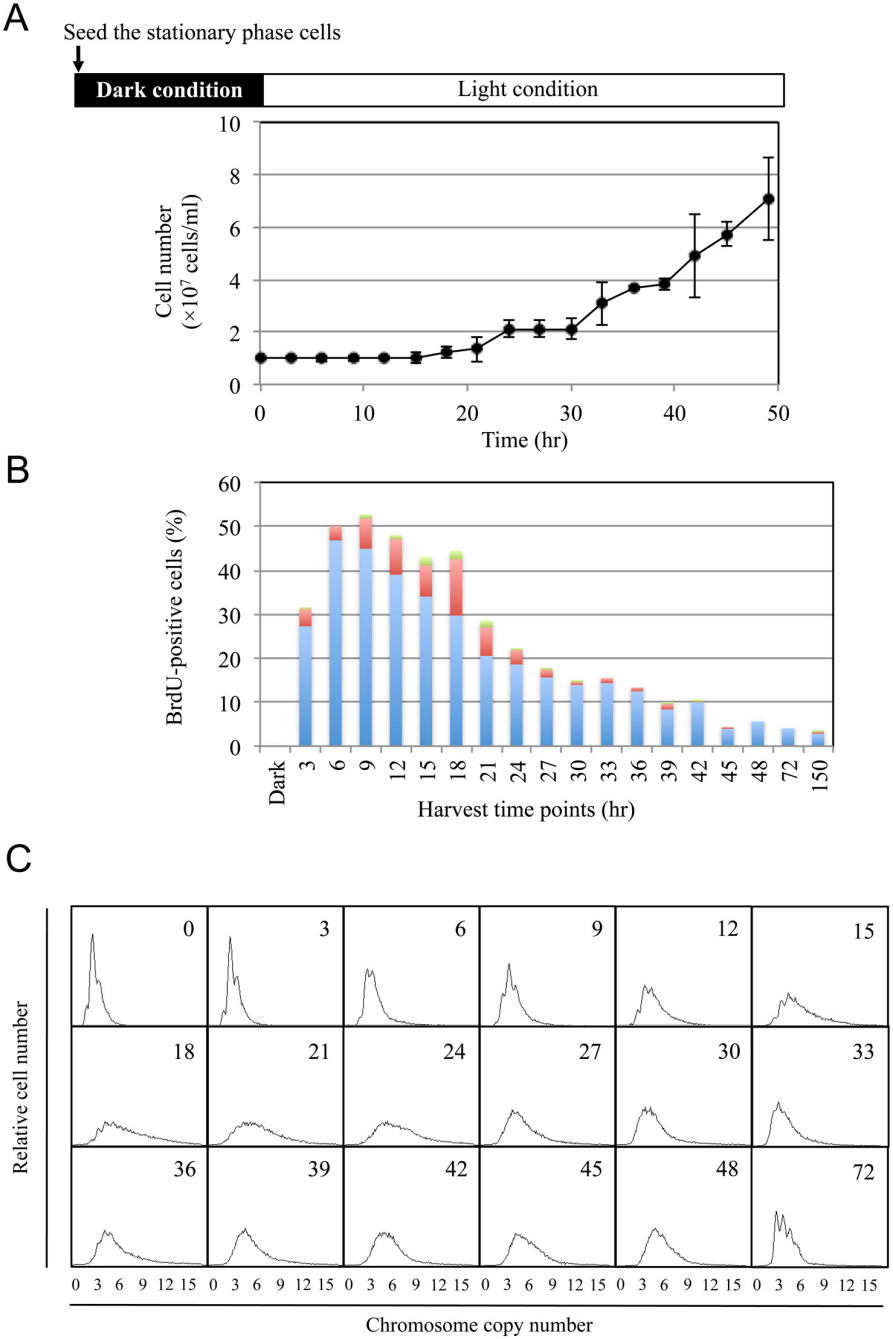
Cell doubling and chromosome copy number post-transfer. (A) Cell numbers post-transfer, which were calculated under a microscope using a bacteria counter (As One Co., Osaka, Japan). (B) The proportion of BrdU-positive cells after exposure to light. *Synechococcus* 7942^TK^ cells were labelled with BrdU for 30 min, harvested at the indicated times, stained with an anti-BrdU antibody, and examined with immunofluorescence microscopy. The proportion of BrdU-positive cells (BrdU-positive cells per 300 cells) is depicted using different colours, with each colour representing a different number of foci per cell: blue = 1 focus, red = 2 foci, green = 3 or more foci per cell. (C) DNA content profiles of *Synechococcus* 7942^TK^ cells. Cells were harvested at each time point and analysed with flow cytometry.

We next compared the frequency of DNA replication in each phase. Because the cell number in *Synechococcus* 7942^TK^ cultures exhibited stepwise doubling rather than a linear increase after transfer to the light condition, the timing of cell division was synchronized under this condition (Fig 2A).

To assess DNA replication, *Synechococcus* 7942^TK^ cultures were treated at various time points (Fig 2B) with 5-bromo-2′-deoxyuridine (BrdU) for 30 min and then examined with immunofluorescence microscopy. As shown in Fig 2A, the cell number doubled between 18 and 24 hr post-transfer. The number of BrdU-positive cells steadily increased upon exposure to light, reaching a maximum 6–9 hr after transfer (Fig 2B). Interestingly, the peak replication time (i.e. the point at which the proportion of BrdU-positive cells was highest) preceded the onset of cell division. Although thymidine kinase was clearly expressed until 120 hr post-transfer (Fig 1C, HA-TK), the number of BrdU-positive cells decreased steadily after the peak replication time. The DNA profile analysis supported this observation. The spike-shaped profile gradually broadened between 0 and 24 hr post-transfer, and the chromosomal copy number increased relative to that in the starting culture (Fig 2C, Panels 0–24 hr post-transfer). After the 24 hr time point, which corresponded to the period just after the onset of cell division, the profile began to exhibit a spiked pattern and eventually regained the spike-shaped pattern at 72 hr post-transfer. We hereafter define the period between the culture transfer and the onset of cell division (~20 hr) as the lag phase. In this phase, intensive DNA replication occurred.

### Accumulation of chromosomal copies in the lag phase and *Synechococcus* 7942 proliferation

**Fig 3:**
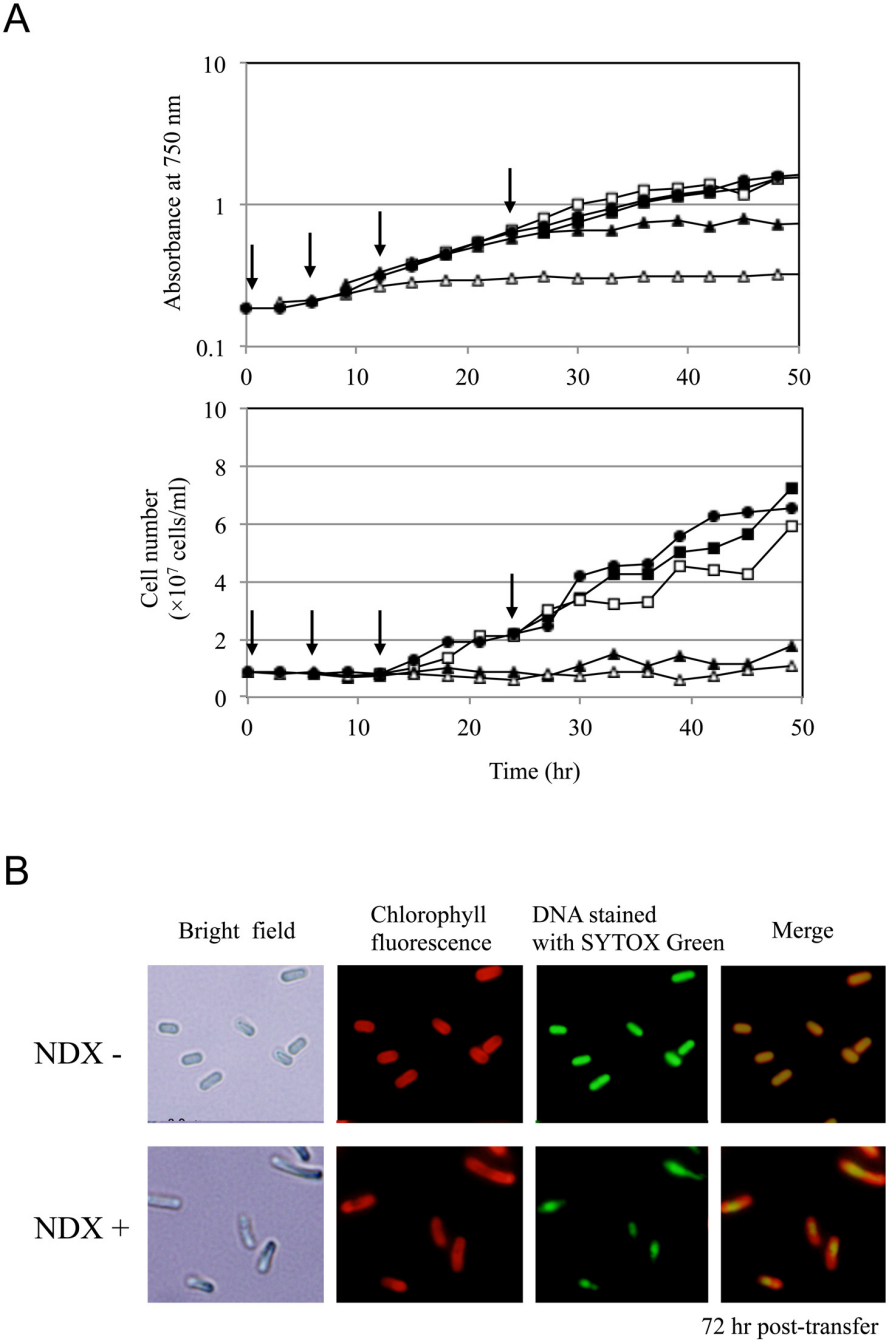
Differences in the effect of NDX at each phase. (A) Growth curve and cell numbers in the presence or absence of nalidixic acid (NDX). NDX (final concentration 3 μg/mL) was added to synchronized *Synechococcus* 7942 wild-type cultures before (open triangles) exposure to light and then at 6 hr (closed triangles), 12 hr (open squares), and 24 hr (closed squares) after exposure. The control batch (without NDX) is indicated by closed circles. Arrows indicate the time points of NDX addition. (B) The effects of NDX addition at 12 hr post-transfer (before the onset of cell division). The cells were harvested at 72 hr post-transfer. Fixed cells were stained with SYTOX Green and examined with microscopy. Bright-field images (bright), SYTOX Green-stained images (SG), fluorescence images of chlorophyll (Chl), and merged images (SYTOX Green-stained and chlorophyll images) (Merge) are shown.

We previously observed that treatment with the DNA gyrase inhibitor nalidixic acid (NDX) inhibited DNA replication in *Synechococcus* 7942 [22, 23]. In order to establish that intensive DNA replication in the lag phase is essential, we compared the effects of NDX. Five sets of synchronized batch cultures were prepared and treated with NDX at 0, 6, 12, and 24 hr after transferring cells to illuminated conditions. While the control culture (without NDX) doubled 12–18 hr post-transfer (Fig 3A, closed circles), the culture treated with NDX before transfer exhibited severe defects in cell growth and cell doubling (Fig 3A, open triangles). However, the effects of NDX treatment gradually decreased as the incubation time increased. Although treatment with NDX at 6 hr post-transfer abrogated cell doubling, the growth rate of the culture was only slightly slower than that of the control (Fig 3A, closed triangles). The addition of NDX at 12 hr post-transfer (open squares) did not affect the growth rate; however, there was a slight defect in cell doubling beginning at 30 hr after transfer. Lastly, the addition of NDX at 24 hr post-transfer (closed squares) had no effect on growth rate or doubling. We compared the morphology of the control cells and the cells treated with NDX at 12 hr post-transfer (Fig 3A, open squares). Cells were harvested, stained with SYTOX Green, and visualized with fluorescence microscopy. Compared to cells in the control culture, the NDX-treated cells were larger, with an aberrant DNA distribution (Fig 3B). These results indicate that the lag phase can be clearly defined by the differences in the effects of NDX on cell proliferation and morphology. Furthermore, they suggest that DNA replication during the lag phase is an essential precursor to proliferation steps. Thus, there is likely a chromosomal copy number threshold that must be attained in order to promote efficient cell division.

### Metabolome profiles of *Synechococcus* 7942 during batch culture

**Fig 4:**
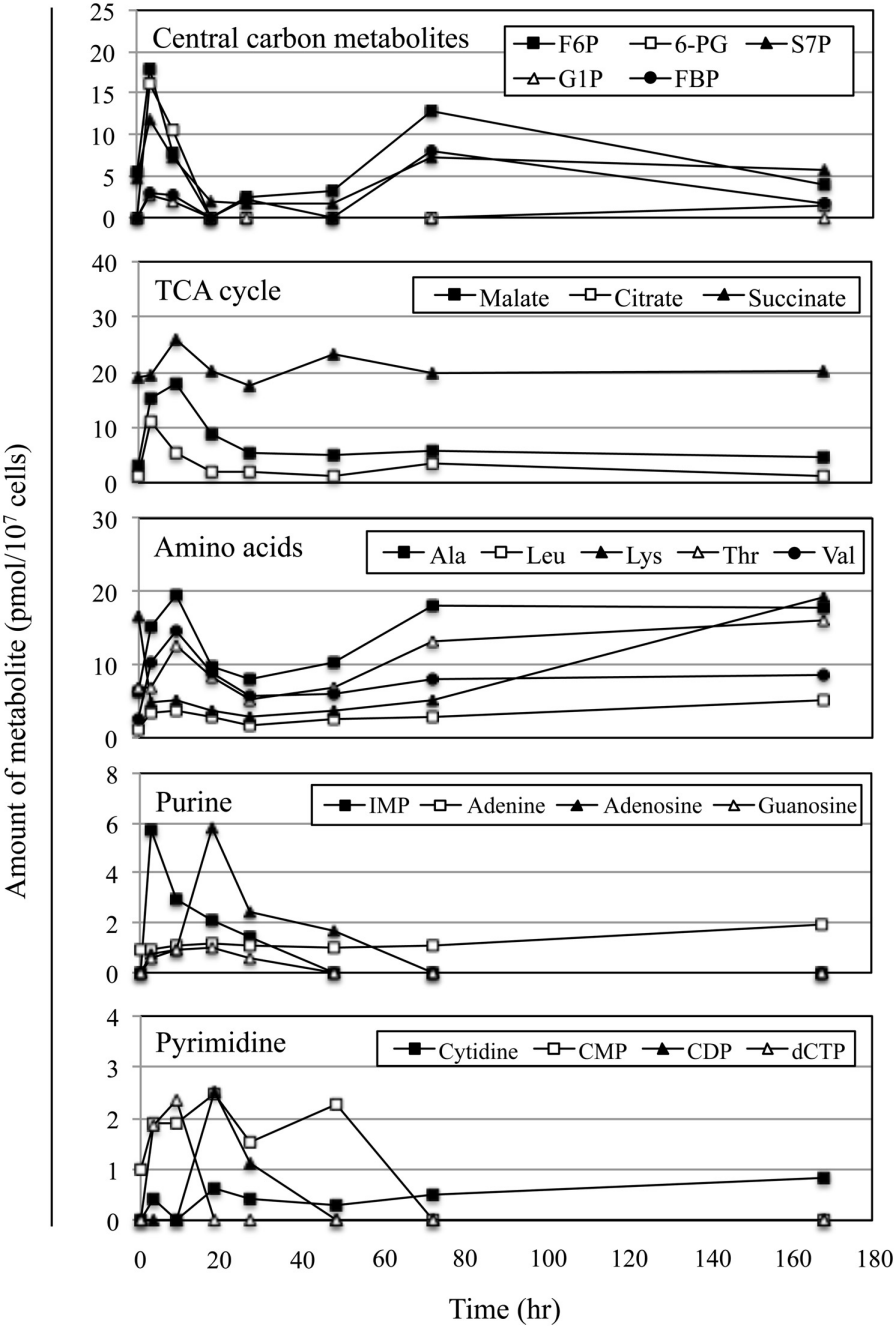
Metabolite profiles of *Synechococcus* 7942 during batch culture. The stationary culture of *Synechococcus* 7942 wild-type was diluted to OD_750_ = 0.2 in fresh BG11 medium. After 18 hr in the dark, cultures were transferred to illuminated conditions (time 0). Samples were harvested at various times points post-transfer. The absolute amounts (pmol per 10^7^ cells) of metabolites were determined at each time point with CE-TOFMS. The data show remarkable changes at each phase in central carbon metabolites, TCA cycle metabolites, amino acids, purines, and pyrimidines. Raw data and additional figures are included in S2 Table, and S2–S5 Figs.

**Fig 5:**
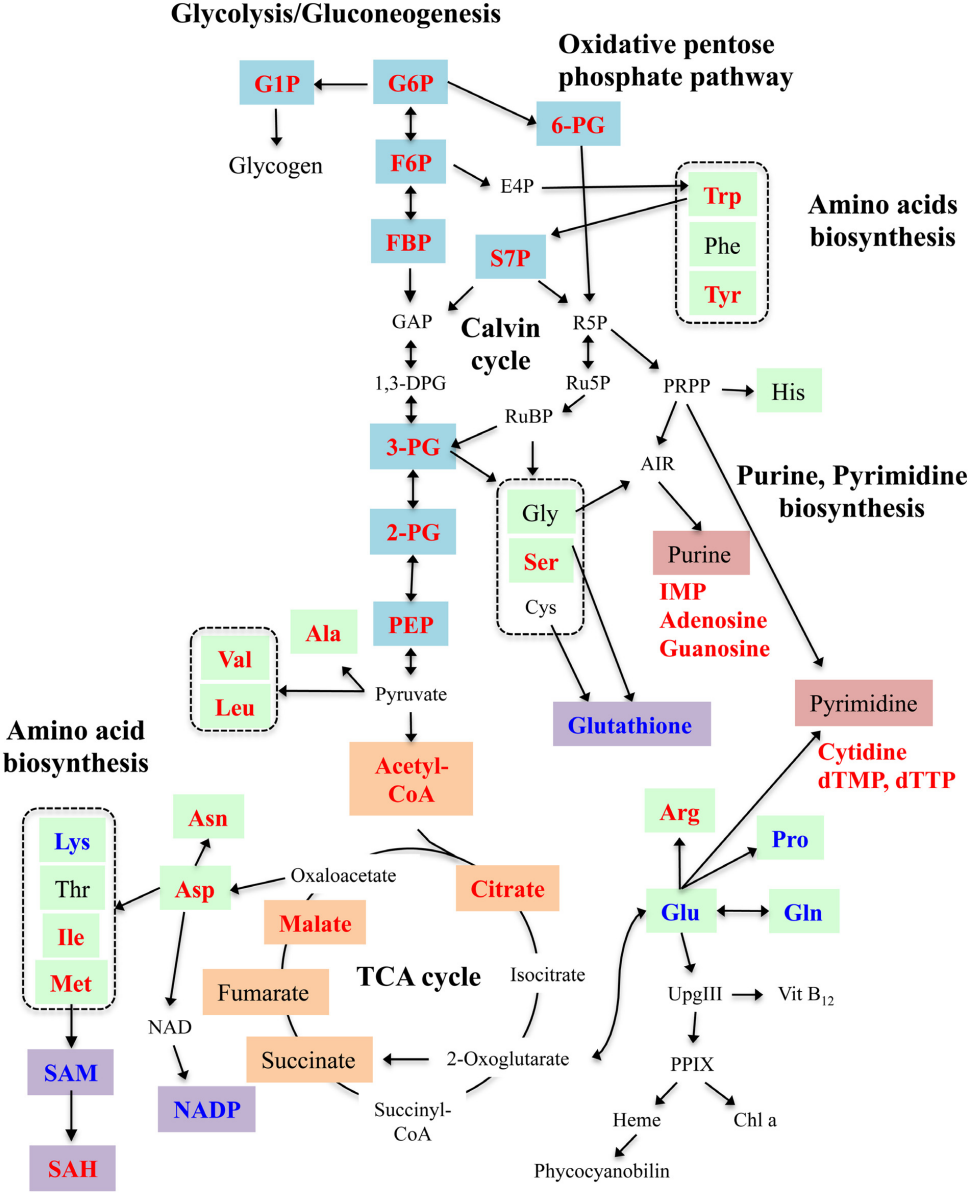
Schematic diagram of central metabolism in *Synechococcus* 7942. The metabolites measured in Fig 4 are highlighted in blue (glycolysis), orange (TCA cycle), green (amino acids), red (purine and pyrimidine), and purple (others: NADP^+^, SAM, SAH, and glutathione). The metabolites that increased (>2) or decreased (<0.5) during lag phase (3 hr or 9 hr after transfer), relative to the amount in the starting culture, are denoted in red and blue fonts, respectively.

To investigate the overall cellular physiology during the lag phase, we compared the metabolome at various time points after transferring cells to illuminated conditions. The timing of cell division in *Synechococcus* 7942 wild-type cells was synchronized, as described above; the cells were harvested at each point (Fig 4) and analysed with CE-TOFMS. The total amounts of 53 metabolites, normalized to the cell number (pmol per 10^7^ cells), are shown in S2 Table, Fig 4, and S2–S5 Figs. The putative metabolic pathway in *Synechococcus* 7942 is depicted in Fig 5.

The level of each metabolite varied throughout the growth phases. Because stationary cultures were diluted and used for the synchronization step, the metabolic state of the starting cultures was similar to those cultured under nutrient- and light-limited conditions. Consequently, upon transfer of these cultures to illuminated conditions, it was necessary for the cells to rapidly produce the various metabolites needed for proliferation. Central carbon metabolites, which are involved in glycolysis, the oxidative pentose phosphate (OPP) pathway, and the TCA cycle (glucose 6-phosphate (G6P), glucose 1-phosphate (G1P), fructose 6-phosphate (F6P), fructose 1,6-bisphosphate (FBP), 6-phosphogluconate (6-PG), and sedoheptulose-7-phosphate (S7P)), accumulated rapidly within 3 hr post-transfer (Fig 4 and S2 Fig). Likewise, the purine precursors, inosine 5′-monophosphate (IMP), adenosine, and guanosine, and the pyrimidine metabolites, cytidine, dTMP, and dTTP, increased transiently during this period (Fig 4 and S4 Fig). These metabolites are essential for energy production and DNA synthesis. These findings are consistent with the initiation of chromosomal replication during the lag phase (Fig 2B). The amounts of these metabolites decreased at 9 hr post-transfer, suggesting that central carbon metabolism and pyrimidine metabolism were obstructed between the 3 hr and 9 hr time periods. However, the accumulation of these molecules was apparently resolved by the activation of metabolic flow downstream of each metabolic pathway. Citrate was the only other molecule with a profile similar to those of the central carbon metabolites: levels increased at 3 hr and then decreased by 9 hr after transfer (Fig 4). In contrast, the levels of five metabolites (glutamate (Glu), proline (Pro), glutathione, NADP^+^, and SAM) transiently decreased at 3 hr but recovered by 9 hr post-transfer (S3 and S5 Figs). The initial reductions in these metabolites might be due to the overflow of carbon metabolites, as mentioned above. As a consequence of their usage, the pools of these molecules might decrease transiently. Several metabolites downstream of the central carbon metabolic pathway (3- and 2-phosphoglycerate (3PG and 2PG), phosphoenolpyruvate (PEP), acetyl CoA, TCA cycle metabolites, and amino acids) as well as ATP and GTP accumulated at 9 hr post-transfer (Fig 4, S2–S4 Figs). The levels of the DNA precursors adenosine and CDP increased transiently at 18 hr post-transfer (Fig 4), which corresponded to the period at which DNA replication rates decreased (Fig 2B). These observations indicate that, in addition to the onset of DNA replication (Fig 2B), there is a dynamic metabolic change during the lag phase, and that activation of the metabolic pathways proceeds in an ordered manner during this period.

At the onset of cell division (18 hr post-transfer) and during the exponential phase (27 hr post-transfer), metabolite levels were depressed, suggesting that metabolic flow progressed smoothly, directly reflecting the rates of cell proliferation. At the beginning of the linear phase (48 hr post-transfer), growth rates began to decrease (Fig 1A) and several metabolites, especially amino acids, started to accumulate (S2 Table, Fig 4, and S3 Fig), indicating that the obstruction of these metabolic pathways had already begun. These responses may act as a trigger for transition from exponential to linear growth phase. A transient accumulation of F6P, FBP, G6P, ATP, and dTTP was observed at the linear phase (72 hr post-transfer) (Fig 4, S2 and S4 Figs). In contrast, the levels of S7P, amino acids, and glutathione continued to increase during the linear phase (Fig 4, S3 and S5 Figs). These metabolic responses may be due to the effects of both light- and partial nutrient-limitation during the linear phase. Because the amounts of these metabolites in the starting culture were low, the levels of these molecules were likely depleted during the dark incubation period after dilution in fresh BG11 medium. Notably, the profile of lysine (Lys) was distinct from those of the other amino acids (Fig 4). The level of Lys immediately decreased post-transfer and gradually increased thereafter, suggesting that the Lys degradation pathway is activated by light irradiation. However, such regulation has yet to be characterized.

## Discussion

**Fig 6:**
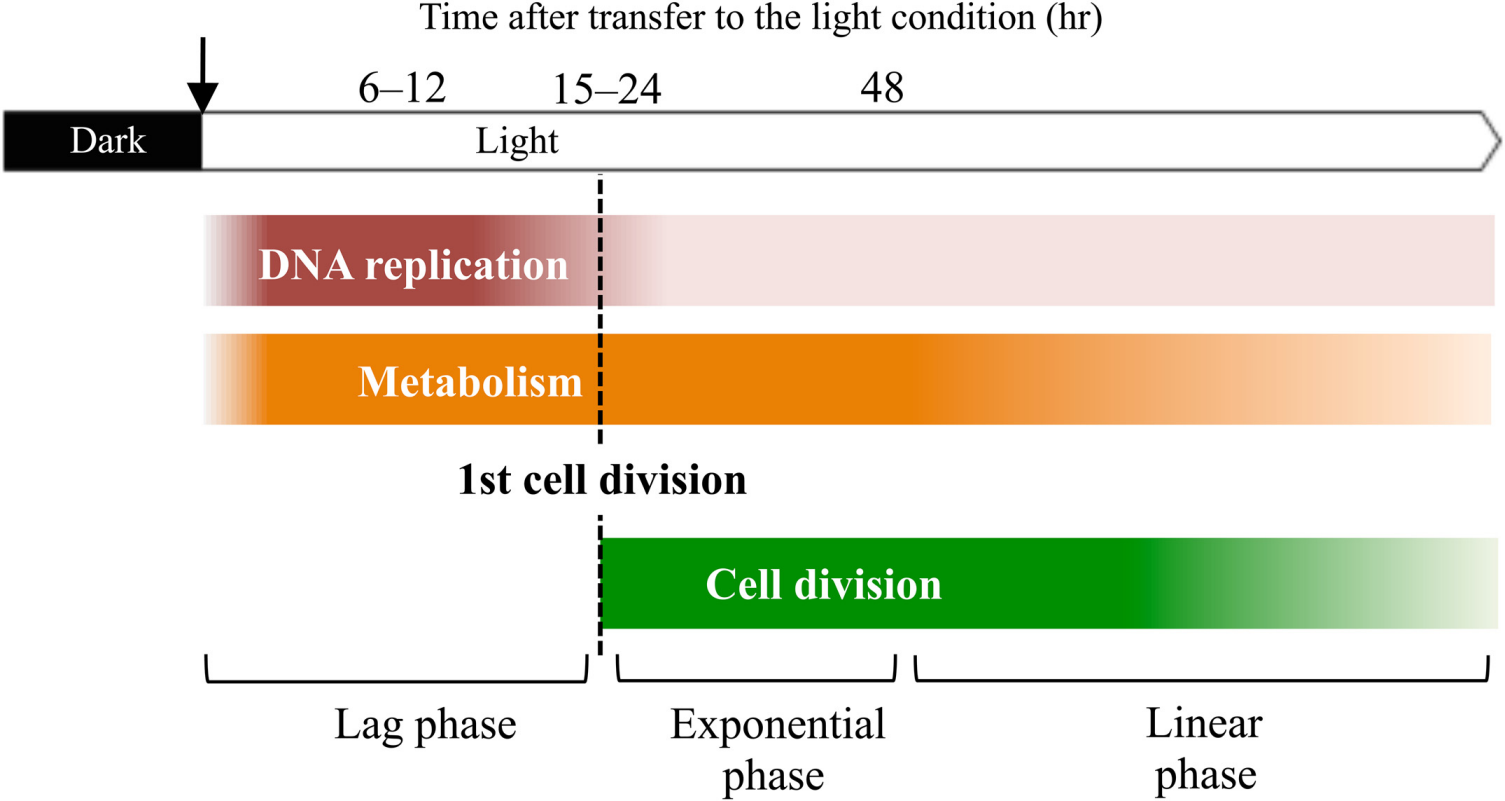
Schematic drawing depicting the growth phases of *Synechococcus* 7942.

We demonstrated that the chromosomal copy number of *Synechococcus* 7942 varied depending on the growth phase. A graphic depiction of the growth phases of *Synechococcus* 7942 during batch culture is shown in Fig 6. In particular, ploidy level and metabolite production were found to increase significantly during the lag phase prior to the onset of cell division, indicating that DNA replication and cell division are not closely coupled in the *Synechococcus* cell cycle. These phenomena are related to regeneration of cells at stationary phase under nutrient- and light-limiting conditions (S1 Table, S1 Fig).

We propose that the chromosomal copy number is determined by the balance between DNA replication and cell growth, including cellular metabolism and cell division. (1) In the lag phase, cells undergo rapid DNA replication before the onset of cell division, which is accompanied by a marked increase in the chromosomal copy number (Fig 2). NDX had a strong effect in the lag phase culture (Fig 3), indicating that the accumulation of chromosomal copies during this period is essential for the initiation of exponential growth. Likewise, a significant, orderly increase in metabolism occurs during the lag phase. However, phosphate content was not found to decrease during the lag phase (S1 Fig), suggesting that *Synechococcus* 7942 utilizes intracellular phosphate during the lag phase. Cells proceeded to the exponential growth phase after generating the necessary chromosomal copies and accumulating sufficient amounts of appropriate metabolites. Our results suggest there is a checkpoint that regulates the transition from the lag phase to the exponential phase. (2) The rate of DNA replication is slower in the exponential phase than in the lag phase, while metabolism proceeds smoothly until the linear phase, reflecting the rapid growth rate. As a result, the chromosome copy number begins to decrease during the exponential phase. In contrast, organisms with a single chromosomal copy, such as *E*. *coli* and *B*. *subtilis*, exhibit high rates of DNA replication during the exponential phase. (3) At the onset of the linear phase, which corresponds to the beginning of light and nutrient limitation, DNA replication and cellular metabolism are markedly reduced; however, cell growth and cell division continue. Hence, the linear phase cultures exhibit a spike-shaped DNA content profile, as shown in Figs 1B and 2D. It is noteworthy that the switch from exponential phase to linear phase seems to be triggered by light limitation rather than phosphate limitation. The principal sigma factor RpoD1 level began to decrease after 48 hr post-transfer (Fig 1C), indicating that the observed changes in the transcription profile reflect the cellular responses described above.

The polyploidy and intensive DNA replication observed in *Synechococcus* 7942 before the first cell division suggests that this species possesses an uncoupled DNA replication system. In *Synechocystis* 6803, multiple chromosome copies are segregated randomly into the two daughter cells during cell division, suggesting that the coordination between chromosome segregation and cell division in *Synechocystis* 6803 is much less stringent than that in *B*. *subtilis* [37].

Similar observations have been made in studies on plastids in plant cells, which arose from a freshwater cyanobacterial lineage [38]. It has been reported that DNA replication in plastids is not tightly linked to nuclear DNA replication or plastid division [39, 40]. Plastids undergo intensive DNA amplification before cell division, which is a fundamental step towards subsequent plastid division. In green plants, such as wheat, barley and pea, the plastid DNA number varies during the developmental stages [39–44], suggesting that the frequency of DNA replication and plastid division changes at each stage. Additionally, in the unicellular algae *Chlamydomonas reinhardtii*, which contain one plastid per cell, the plastid DNA replicates multiples times before plastid division [45]. In addition, phosphate limitation strongly affects the copy number of plastid DNA in *Chlamydomonas* [46]. Our findings are expected to not only contribute to an improved mechanistic understanding of cyanobacterial growth physiology, but also to provide insights into the evolution of plastids.

## Supporting Information

S1 Fig The amount of inorganic phosphate and the expression of genes involved in *Synechococcus* 7942 phosphate uptake in a batch culture. (A) Growth curve of *Synechococcus* 7942 after transfer to illuminated conditions. (B) The amount of inorganic phosphate during batch culture at each time point was measured using Malachite Green Phosphate Assay Kit (BioAssay Systems co., CA, USA). (C) RNA blot analyses of phosphate binding protein-encoding transcripts (*sphX*: Synpcc7942_2445 and *pstS*: Synpcc7942_2444) in *Synechococcus* 7942, with ethidium bromide-stained 16S rRNA as the loading controls. Culture aliquots were withdrawn for extraction of total RNA before (time 0) and at the indicated times after transfer to light conditions. Five μg of total RNA was loaded onto each lane.
(TIF)

S2 Fig Profiles of central carbon and TCA metabolites in batch culture. The absolute amounts (pmol per 10^7^ cells) of metabolites post-transfer were determined at each time point with CE-TOFMS. The data for the central carbon (A) and TCA (B) metabolites are shown. The raw data is included in S2 Table.
(TIF)

S3 Fig Profiles of amino acids in batch culture. (TIF)

S4 Fig Profiles of purine and pyrimidine phosphate in batch culture. (TIF)

S5 Fig Profiles of NADP, SAM, SAH, and glutathione in batch culture. (TIF)

S1 Table Composition of BG11 medium before and after 1 week of Synechococcus 7942 cultivation. (DOCX)

S2 Table Raw data from the metabolite profiles of Synechococcus 7942 in batch culture. (XLSX)
