# Supplementary figures and images for: Intensive DNA Replication and Metabolism during the Lag Phase in Cyanobacteria
Source: PLoS One. 2015 Sep 2;10(9):e0136800. doi: 10.1371/journal.pone.0136800 (PMC4558043; doi:10.1371/journal.pone.0136800)

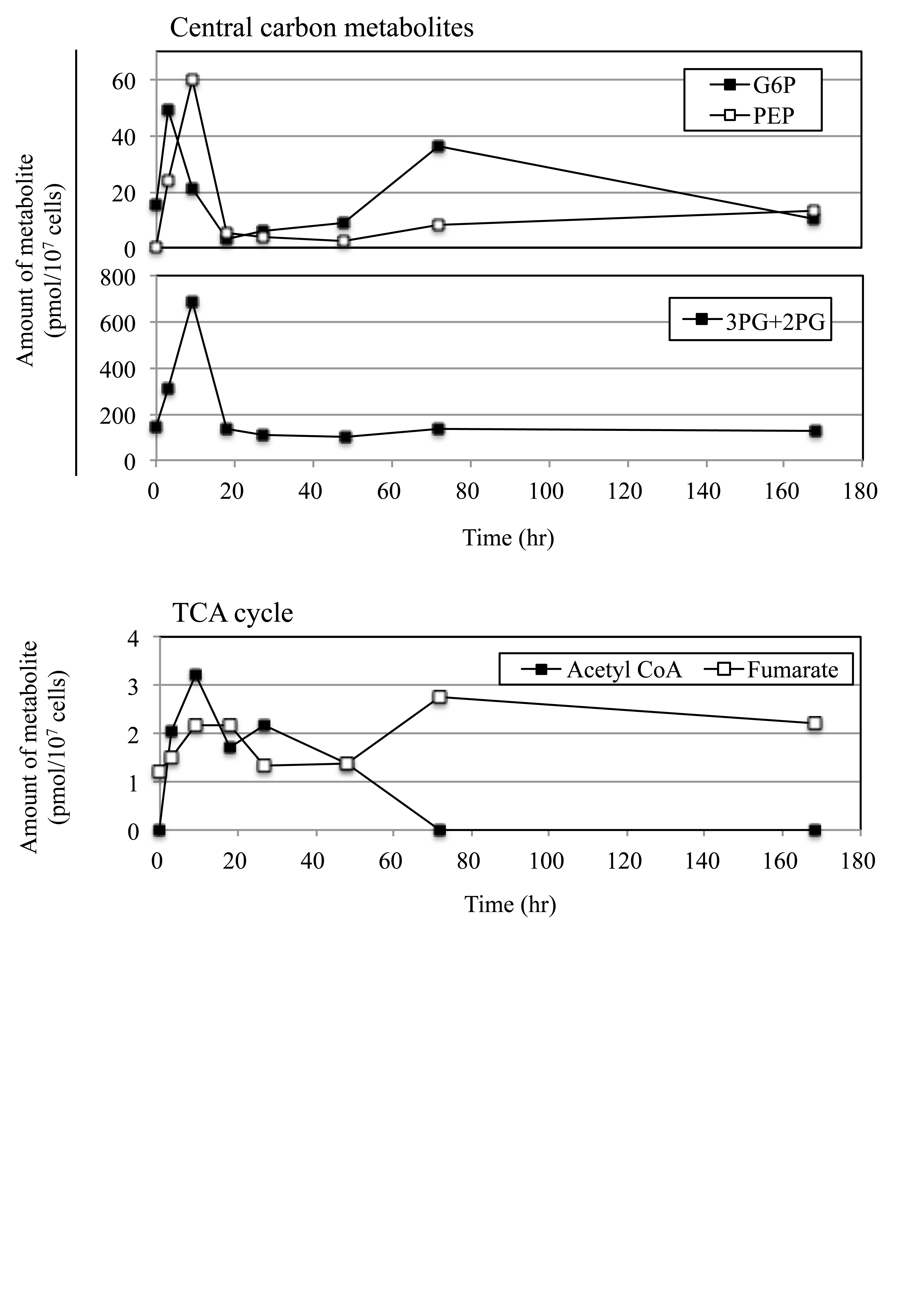

Supplement: S2 Fig — The absolute amounts (pmol per 107 cells) of metabolites post-transfer were determined at each time point with CE-TOFMS. The data for the central carbon (A) and TCA (B) metabolites are shown. The raw data is included in S2 Table. (TIF) [file pone.0136800.s002.tif]

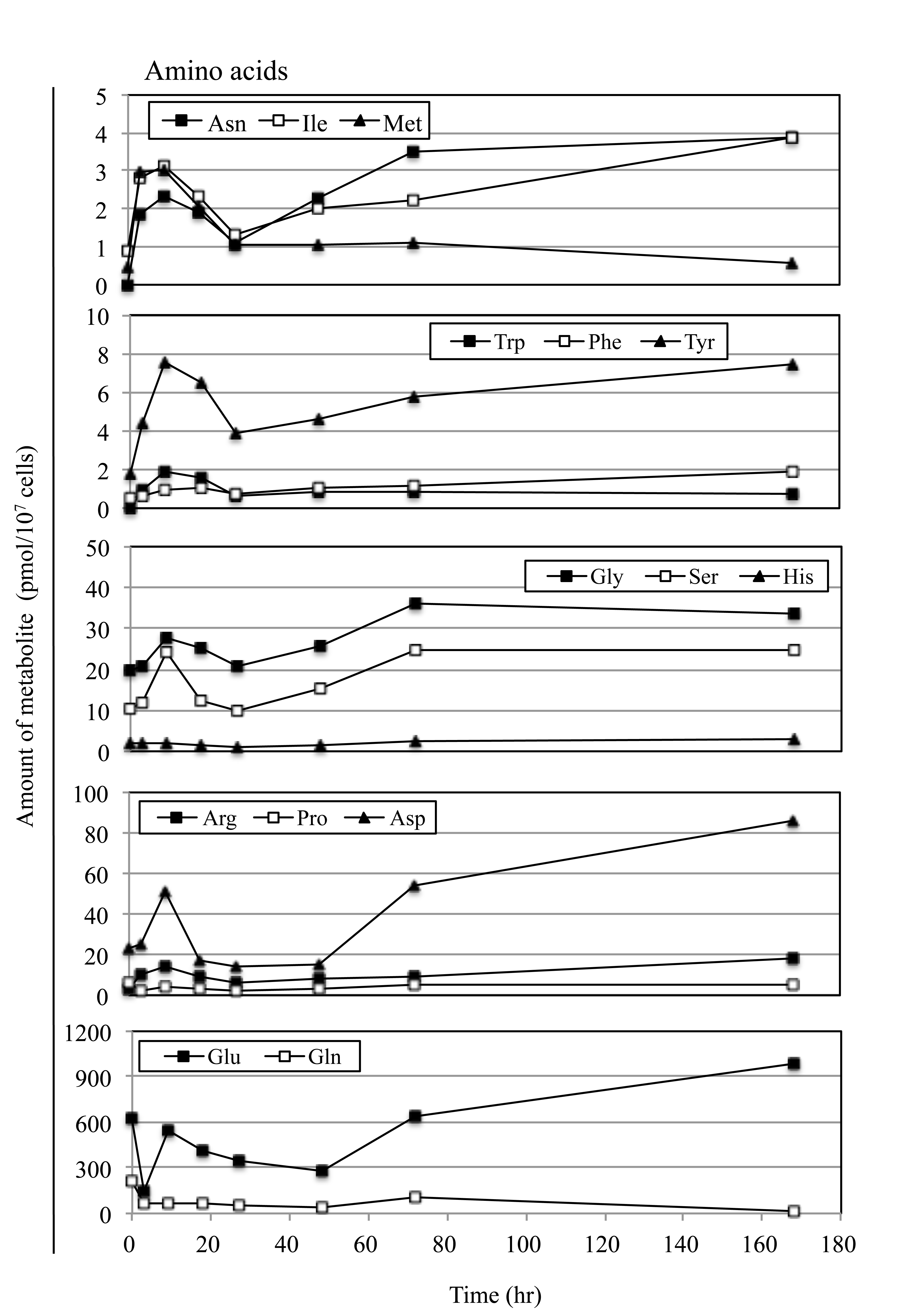

Supplement: S3 Fig — (TIF) [file pone.0136800.s003.tif]

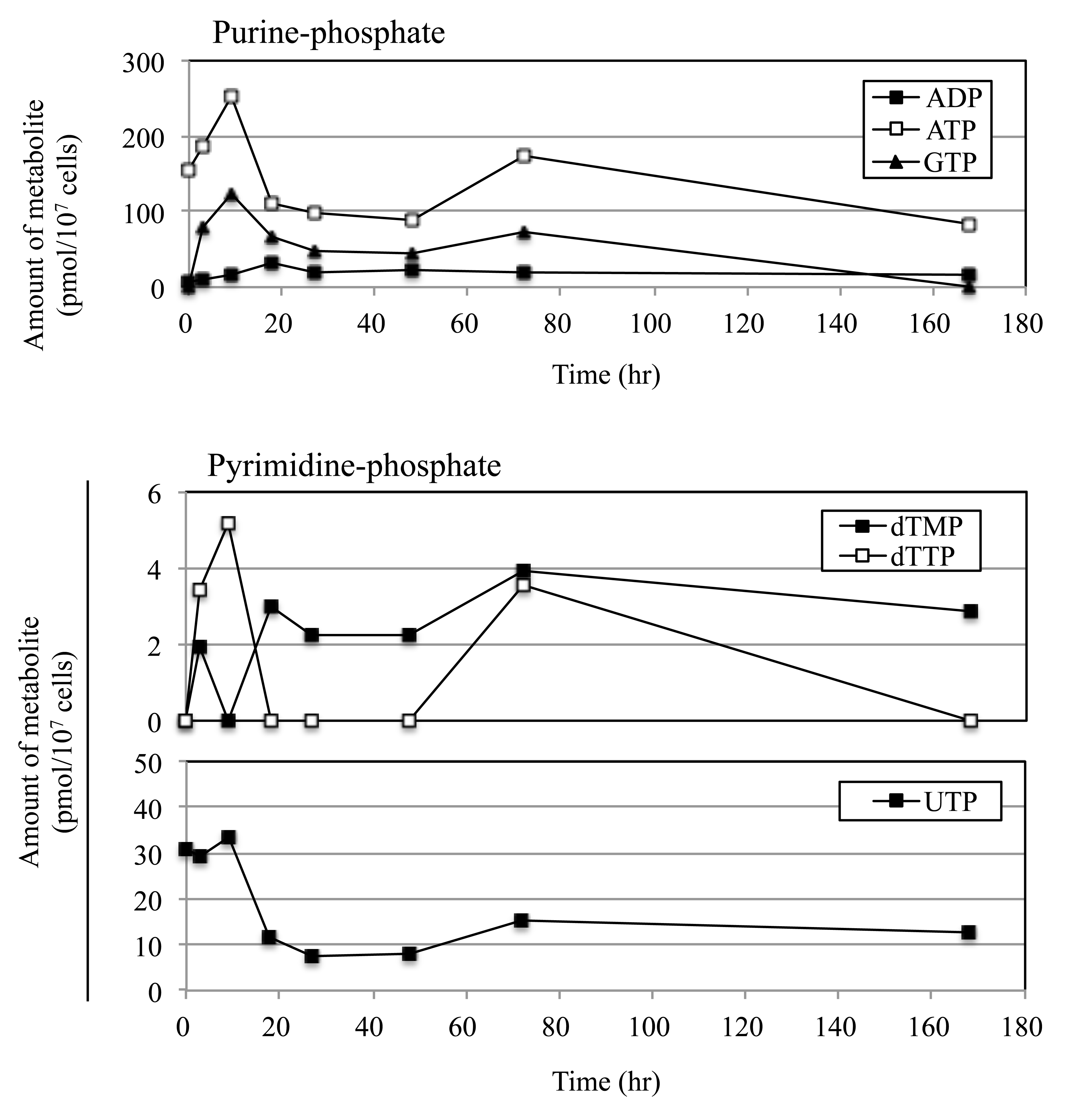

Supplement: S4 Fig — (TIF) [file pone.0136800.s004.tif]

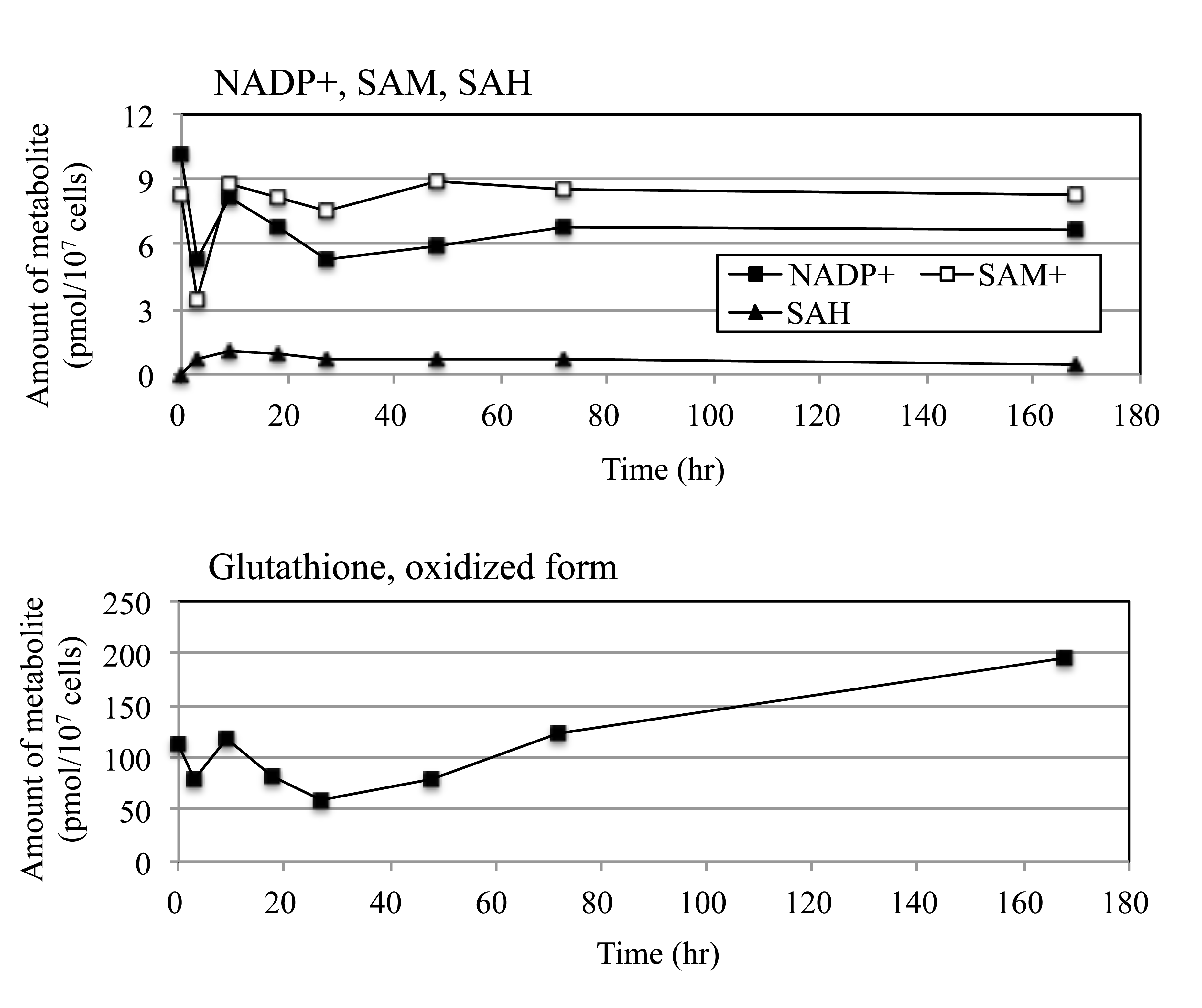

Supplement: S5 Fig — (TIF) [file pone.0136800.s005.tif]
