# Supplementary material for: Intensive DNA Replication and Metabolism during the Lag Phase in Cyanobacteria
Source: PLoS One. 2015 Sep 2;10(9):e0136800. doi: 10.1371/journal.pone.0136800 (PMC4558043; doi:10.1371/journal.pone.0136800)
Supplement: S1 Table — (DOCX) [file pone.0136800.s006.docx]

**S1 Table.** **Composition of BG11 medium before and after 1 week of *Synechococcus* 7942 cultivation.**

Before After 1-week Ratio

cultivation of cultivation (After / Before)

*Anion*

Nitrate (NO_3_^-^) 2400 2100 0.88

Sulphate (SO_4_^2-^) 32 18 0.56

Chlorine (Cl^-^) 24 20 0.83

**Phosphate (PO_4_^3-^) 14 <0.5 <0.04**

*Cation*

Sodium (Na^+^) 780 840 1.08

**Potassium (K^+^) 12 4.5 0.38**

Calcium (Ca^2+^) 10 9.4 0.94

Magnesium (Mg^2+^) 7.2 5.8 0.81

**Iron (Fe^3+^) 1.1 0.02 0.02**

Manganese (Mn^2+^) 0.51 0.52 1.02

The concentration of all compounds is expressed as mg/L. After 1-week of *Synechococcus* 7942 cultivation, the concentration of ions that reduced to less than half of that in fresh BG11 medium are shown in bold.
